# The Question of Senior Partnership

**Published:** 1912-10-26

**Authors:** 


### THE QUESTION OF SENIOR PARTNERSHIP.

A good many sanatoriums will now have received their first patient benefiting under the National Insurance Act and sent in by the local Insurance Committee. The preliminaries leading up to this result have not, however, been got through without some misgivings and heart-burnings. Old supporters and committeemen, often not favourably disposed politically to the new order of things, have felt with justice that they, who started the institution and stood the expense and general " racket " of establishing it free of debt and in good going order, should not be swamped in the management by newcomers to the vineyard, and newcomers, moreover, on particularly easy terms; for " sanatorium benefit " rests and counts upon a foundation of charitable effort, the Local Government Board's policy seeming at present to be marked by a desire to leave any taking of risks to private enterprise or to bodies altogether decentralised. Changes are bound to be noticeable, and possibly existing committee members, while feeling it right that their institution should have as large as possible a field of usefulness, may find not quite pleasant the idea of taking in a rather different class of patient from that originally contemplated. A favourite social stratum to provide for used to be the one which could afford to supplement charity a little out of its own pocket, whereas now almost any member of the proletariat is eligible. For these and many other little reasons, men and women who would in no sense subscribe to the famous aphorism, "Any change, at any time, for any purpose, is to be deprecated," may be pardoned some tetchiness at this period of transformation in the hospital world.

The question is, What can be done or said to allay these feelings and promote harmony of the old with the new controlling elements? In the first place, those who are and should remain the senior partners may comfort themselves with the thought that there is, after all, a law of property; trustees and the like responsible officials cannot, for a generation at any rate, suffer much real displacement. Nevertheless, it stands to common sense that a stiff attitude would be very unwise. Widespread social amelioration is a most praiseworthy aim, in comparison with which personal susceptibilities cannot weigh for an instant. And this amelioration entails democratic support, and therefore some measure of democratic control, which—in things medical at any rate, as Sir William Collins and others maintain—is not really inimical to intellect and the things of the mind, but to privilege and a conventionally aristocratic spirit. It is remarkable, too, and perhaps a little depressing, to observe how rapidly the passage of time and the responsibilities of office—in effect, the " world's slow stain "—modify the most energetic reformers. Oft-times King Stork has not been long on the throne before it gets hard to distinguish his reign from that of his predecessor. And even when his impetus remains unslowed, posterity is not a severe critic at all: it finds nothing much to be astonished at. But contemporaries do; and the prospect of becoming unpopular with constituents by imposing a threepenny rate is doubtless now giving pause to a good many ardent spirits.

A word as to the *personnel* of a public sanatorium. The essentials are: first, a representative managing committee, with a few active members. Prominent among these latter is, secondly, a medical secretary with two great qualifications—the one tact, the other extensive practice in diagnosing consumption; a junior consultant will not do. Undoubtedly the official must now be paid. Lastly, an able and versatile resident superintendent, well up with the literature of tuberculosis and inspiring respect amongst patients professionally, yet a fine administrator. Given such a staff, all that remains is to give it plenty of backing and a free hand.

